# A refinable three-parameter equation for phenomenological absorption in quantitative electron microscopy – determining the equation

**DOI:** 10.1107/S160057672500545X

**Published:** 2025-09-04

**Authors:** Philip N. H. Nakashima, Tianyu Liu, Andrew E. Smith, Laure Bourgeois

**Affiliations:** a Department of Materials Science and Engineering Monash University Victoria3800 Australia; b Institute of Multidisciplinary Research for Advanced Materials Tohoku University Sendai980-8577 Japan; c School of Physics and Astronomy Monash University Victoria3800 Australia; d Monash Centre for Electron Microscopy Monash University Victoria3800 Australia; University of Warsaw, Poland

**Keywords:** phenomenological absorption, quantitative convergent-beam electron diffraction, differential QCBED, quantitative transmission electron microscopy, electron scattering calculations, inelastic scattering factors

## Abstract

A three-term equation for phenomenological absorption of electron beams in materials is derived for use in quantitative transmission electron microscopy simulations. This is motivated by differential quantitative convergent-beam electron diffraction (QCBED) using CBED patterns that have not been electron-optically energy filtered. As a starting point, this three-parameter function reproduces inelastic scattering factors generated by the ubiquitous *ATOM* subroutine of Bird & King [*Acta Cryst.* (1990), A**46**, 202–208] to within a few per cent, spanning elements *Z* = 1 to 98, Debye–Waller parameters from *B* = 0.05 Å^2^ to *B* = 2.0 Å^2^, scattering angles from *s* = 0 Å^−1^ to *s* = 6.0 Å^−1^ and electron energies from *E*_0_ = 1 keV to *E*_0_ = 1 MeV. As such, it is applicable to zero-energy-loss electron-optically filtered pattern matching for which the Bird and King *ATOM* subroutine was designed. Crucially, the coefficients of the three terms in the present equation can be refined to produce inelastic scattering factors with differing local and non-local contributions, which are better suited to unfiltered differential QCBED pattern matching.

## Abstract 2

A three-parameter function is derived empirically, based on the inelastic scattering factors of Bird & King [*Acta Cryst.* (1990), A**46**, 202–208], for incorporating atom-localized and non-local phenomenological absorption into quantitative electron diffraction and imaging, with and without electron-optical energy filtering.

## 1. Introduction

The highly dynamic process of electron scattering from even the thinnest material samples complicates the accurate simulation of electron microscope images and diffraction patterns. The problem is considerably simplified if an electron-optical energy filter is used to exclude almost all of the inelastically scattered electrons. With the advent of electron energy-loss spectrometers and energy-filtered imaging with transmission electron microscopes (TEMs) in the late 1980s and early 1990s, Bird & King (1990 ▸) met the need for a description of absorption due to thermal diffuse scattering (TDS) in elastic electron scattering simulations through their *ATOM* subroutine.

Phenomenological absorption collectively accounts for all electrons that are lost from the signal recorded in a TEM that is to be matched by simulations, irrespective of whether the intensities correspond to an image of the specimen or a diffraction pattern from it. In the context of quantitative transmission electron microscopy, the matching of an experimental image or diffraction pattern with a simulated one allows fundamental materials properties to be accurately measured via refinement as variable parameters in pattern-matching processes. Electrons lost from the signal to be matched can include those that have lost energy due to

(i) core–shell excitations,

(ii) bremsstrahlung,

(iii) plasmon excitations and

(iv) TDS arising from phonon excitation.

Electron energy filters allow the removal of (i), (ii) and (iii) above but not (iv) due to the sub-0.1 eV losses associated with TDS. The lack of an energy filter means that all of these components will be present.

The ‘10% rule’ – *i.e.* that the imaginary absorptive part of a structure factor is approximately one-tenth the elastic com­ponent (Hashimoto *et al.*, 1962 ▸; Hall & Hirsch, 1965 ▸; Humphreys & Hirsch, 1968 ▸; Allen & Rossouw, 1990 ▸; Bird & King, 1990 ▸; Rossouw *et al.*, 1990 ▸) – was used widely in early work that attempted to incorporate the effects of absorption into quantitative analyses of electron diffraction and imaging. The growing need for more accurate descriptors of inelastic electron scattering (and thus absorption) led to the analysis of quantum mechanical atomic models and tabulations of inelastic scattering factors resulting from them (*e.g.* Radi, 1970 ▸). Meanwhile, experimental studies were carried out to examine the effects of absorption on individual reflections in electron diffraction patterns (Ishida *et al.*, 1975 ▸; Buxton & Loveluck, 1977 ▸; Ichimiya & Lehmpfuhl, 1988 ▸; Rossouw *et al.*, 1990 ▸). Analytical approaches that were based on a two-beam treatment of dynamical scattering (*e.g.* Hall & Hirsch, 1965 ▸; Radi, 1970 ▸) were extended to many-beam treatments in succeeding studies (*e.g.* Bird & King, 1990 ▸; Rossouw *et al.*, 1990 ▸; Rossouw & Miller, 1993 ▸).

Coincident with the advent of computer-automated quantitative convergent-beam electron diffraction (QCBED), Bird & King (1990 ▸) identified and satisfied the need for a subroutine that could be used in computer programming to provide a more complete *n*-beam analytical treatment of phenomenological absorption. Their resulting *ATOM* subroutine became an intrinsic component of many QCBED algorithms (Bird & Saunders, 1992 ▸; Zuo, 1993 ▸; Deininger *et al.*, 1994 ▸; Midgley & Saunders, 1996 ▸; Tsuda & Tanaka, 1999 ▸; Holmestad *et al.*, 1999 ▸; Saunders *et al.*, 1999*a* ▸; Saunders *et al.*, 1999*b* ▸; Streltsov *et al.*, 2003 ▸; Friis *et al.*, 2004 ▸; Zuo, 2004 ▸; Nakashima, 2007 ▸; Nakashima & Muddle, 2010*b* ▸; Sang *et al.*, 2010 ▸) as well as a variety of other TEM image and diffraction pattern simulation programs (Peng & Whelan, 1992 ▸; Pennycook & Jesson, 1992 ▸; Gajdardziska-Josifovska *et al.*, 1993 ▸; Zuo & Spence, 1993 ▸; Twesten *et al.*, 1997 ▸; Jansen *et al.*, 1998 ▸; Tabira *et al.*, 2000 ▸; Cosgriff & Nellist, 2007 ▸; Tsuda *et al.*, 2007 ▸; Neish *et al.*, 2013 ▸; Hosokawa *et al.*, 2015 ▸; Palatinus *et al.*, 2015 ▸; Shao & Zuo, 2017 ▸).

Since the *ATOM* subroutine of Bird & King (1990 ▸), which applies an Einstein model of TDS in determining inelastic scattering factors, numerous other theoretical and experimental treatments of absorption potentials have emerged in the quest for improved accuracy (*e.g.* Weickenmeier & Kohl, 1991 ▸; Anstis, 1996 ▸; Peng, 1997 ▸; Weickenmeier & Kohl, 1998 ▸; Saunders *et al.*, 1999*a* ▸; Allen *et al.*, 2001 ▸; Ishizuka, 2002 ▸; Zuo, 2004 ▸; Neish *et al.*, 2013 ▸; Allen *et al.*, 2015 ▸; Pennington *et al.*, 2018 ▸; Thomas *et al.*, 2024 ▸). Even so, the *ATOM* subroutine remains very widely used as a good approximation to absorption that is also easy to implement.

In the past two decades, a new approach to QCBED has been developed that does not require the use of energy-filtering electron optics (Nakashima, 2007 ▸; Nakashima & Muddle, 2010*b* ▸; Nakashima *et al.*, 2011 ▸; Nakashima, 2012 ▸; Nakashima, 2017 ▸; Peng & Nakashima, 2017 ▸; Peng & Nakashima, 2019 ▸; Nakashima, 2019 ▸; Peng & Nakashima, 2021 ▸; Tan *et al.*, 2024 ▸). The new approach encompasses development of two different types of differential techniques that involve (*a*) differentiation with respect to specimen thickness (Nakashima, 2007 ▸; Nakashima & Muddle, 2010*b* ▸) and (*b*) differentiation with respect to scattering angle (Nakashima & Muddle, 2010*a* ▸; Nakashima & Muddle, 2010*b* ▸). In the development of these techniques, it was shown that both types of differentiation result in almost complete annulment of the diffuse slowly varying inelastic signal in CBED patterns that contributes a significant background deleterious to QCBED pattern-matching measurements of bonding-sensitive structure factors.

With the advent of differential QCBED, three options have become available for conducting QCBED pattern-matching refinements. These are listed in the left-most column of Table 1 ▸. The different inelastic contributions to CBED intensities, according to what is known of the angular distribution of inelastically scattered electrons in CBED patterns (*e.g* Bird & King, 1990 ▸; Nakashima & Muddle, 2010*a* ▸; Nakashima & Muddle, 2010*b* ▸; Egerton, 2011 ▸; Dwyer, 2014 ▸; Egoavil *et al.*, 2014 ▸), are listed in the top row of the table. Table 1 ▸ shows that the three different modes of QCBED will contain different inelastic contributions.

In conventional QCBED with energy filtering, the standard practice was to remove the TDS diffuse background remaining in CBED patterns by measuring the magnitude of this background surrounding each reflection disc and subtracting a constant average value from each disc individually (*e.g.* Streltsov *et al.*, 2003 ▸). This was reasonable as the TDS background is very slowly varying. The calculated patterns in conventional QCBED included the Bird & King (1990 ▸) inelastic scattering factors from the *ATOM* subroutine.

Differential QCBED with energy filtering does not need the diffuse TDS background to be subtracted in pre-processing of the CBED pattern being matched as this is differentiated out. The inelastic scattering factors from the *ATOM* subroutine (Bird & King, 1990 ▸) can be used in the pattern-matching calculations since the rocking curve to be matched is only affected by TDS.

In the case of differential QCBED without energy filtering, the effect on the rocking curve signal is different due to the contribution of plasmon-loss electrons, which mimics the elastic intensity distribution, albeit with a slight amount of blurring (Egerton, 2011 ▸). This is a significant contribution as plasmon losses constitute the largest component of inelastic scattering in an electron energy-loss spectrum, especially at the specimen thicknesses QCBED typically requires (500–4000 Å). In this case, inelastic scattering factors need to be included that are different from those of Bird and King or similar TDS-based models (*e.g.* Thomas *et al.*, 2024 ▸).

To furnish this requirement, a parametrized function has been developed that, at its base, approximates the *ATOM* subroutine of Bird & King (1990 ▸). This has the following form:
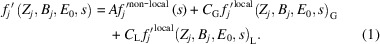
The coefficients *A*, *C*_G_ and *C*_L_ are refinable by QCBED, and the inelastic scattering factor for the *j*th atom in the unit cell, *f_j_′*, depends on the atom’s atomic number (*Z_j_*), its Debye–Waller parameter at the relevant temperature (*B_j_*), the incident electron energy (*E*_0_) and *s* [*s* = sin(θ)/λ] as inputs. The first term summarizes the non-local contributions in the form of a Dirac delta function, and the second and third terms describe the atom-localized contributions – the second term being Gaussian (with coefficient *C*_G_) and the third term being Lorentzian (with coefficient *C*_L_).

The first term is usually redundant because it is automatically taken care of when calculated CBED intensities are normalized to the experimental intensities. However, it is included in the present formulation because one can measure the incident electron beam intensity and fix it as the normalizing factor, and then refine the *A*, *C*_G_ and *C*_L_ parameters to measure the relative local versus non-local contributions. The requirement for separate Gaussian and Lorentzian terms for the atom-localized contributions comes from the form of the Bird & King (1990 ▸) inelastic scattering factors embodied in the *ATOM* subroutine. The aim of the present work is to find functions for 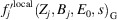 and 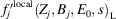 so that, when *A* = 0 and *C*_G_ = *C*_L_ = 1, equation (1) will reproduce the Bird & King (1990 ▸) inelastic scattering factors as a starting point but is applicable to both forms of QCBED employing electron-optical zero-loss energy filtering.

For the case of differential QCBED without energy filtering, the coefficients in equation (1) can be refined so that inelastic scattering factors that depart from those of Bird & King (1990 ▸) can be applied in the presence of the additional plasmon contribution to the rocking curves being pattern matched. It is expected that the coefficients for the Gaussian and Lorentzian terms, *C*_G_ and *C*_L_, will no longer be equal in this case (as well as departing from unity) because the angular dependence of the magnitude of the plasmon contribution to the differential rocking curve will be different from that of the TDS contribution. This can be inferred from the way in which the diffuse background in an unfiltered CBED pattern falls off much more rapidly as a function of angle from the central beam than the TDS background in a zero-loss filtered CBED pattern, which is much more uniform throughout the pattern.

## 2. Analysis

An empirical determination of the function summarized by equation (1) is now described in detail. Considering not only Bird & King (1990 ▸) but also other descriptions of absorption (*e.g.* Humphreys & Hirsch, 1968 ▸; Ishida *et al.*, 1975 ▸; Ichimiya, 1985 ▸; Ichimiya & Lehmpfuhl, 1988 ▸; Peng *et al.*, 1998 ▸), the inclusion of a Gaussian term appears necessary. Even the most primitive approximations used prior to the work of Bird & King (1990 ▸) suggest this because they set *V***_g_**′ = 0.1*V***_g_** (Hashimoto *et al.*, 1962 ▸; Hall & Hirsch, 1965 ▸; Humphreys & Hirsch, 1968 ▸; Allen & Rossouw, 1990 ▸; Bird & King, 1990 ▸; Rossouw *et al.*, 1990 ▸). Here, *V***_g_** is the structure factor of the crystal potential for scattering vector **g** in units of volts and *V***_g_**′ is the associated absorption (inelastic scattering) factor. Following this crude approximation, and given
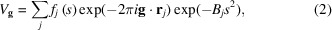
where *f_j_*(*s*) is the atomic scattering factor for the *j*th atom and **r**_*j*_ is its position in the unit cell, then,
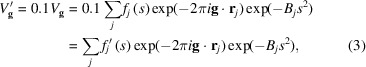
and so,
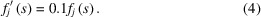
Given *f_j_*(*s*) is very well approximated by a sum of Gaussians in *s* (*e.g.* Fox *et al.*, 1989 ▸; Peng *et al.*, 1996 ▸; Brown *et al.*, 2006 ▸; Colliex *et al.*, 2006 ▸), it follows that *f_j_′*(*Z*_*j*_, *B*_*j*_, *E*_0_, *s*) would include a significant Gaussian term as a function of *s*.

Bird & King (1990 ▸) and a number of other investigators (*e.g.* Humphreys & Hirsch, 1968 ▸; Ishida *et al.*, 1975 ▸; Ichimiya, 1985 ▸; Ichimiya & Lehmpfuhl, 1988 ▸) suggested that *f_j_′*(*Z*_*j*_, *B*_*j*_, *E*_0_, *s*) may be Lorentzian in form in its tail region (higher values of *s*), so the approach adopted in this work is to test sums of Gaussians and Lorentzians in fitting a range of output from the *ATOM* subroutine.

The present aim is to mimic the model of Bird & King (1990 ▸), *f*_*j*_′^B&K^(*Z*_*j*_, *B*_*j*_, *E*_0_, *s*), by a single function, *f*_*j*_′^local^(*Z*_*j*_, *B*_*j*_, *E*_0_, *s*), so that *f*_*j*_′^local^(*Z*_*j*_, *B*_*j*_, *E*_0_, *s*) ≃ *f*_*j*_′^B&K^(*Z*_*j*_, *B*_*j*_, *E*_0_, *s*) and *f*_*j*_′^local^(*Z*_*j*_, *B*_*j*_, *E*_0_, *s*) = *C*[*f*_*j*_′^local^(*Z*_*j*_, *B*_*j*_, *E*_0_, *s*)_G_ + *f*_*j*_′^local^(*Z*_*j*_, *B*_*j*_, *E*_0_, *s*)_L_], where *f*_*j*_′^local^(*Z*_*j*_, *B*_*j*_, *E*_0_, *s*)_G_ and *f*_*j*_′^local^(*Z*_*j*_, *B*_*j*_, *E*_0_, *s*)_L_ are the Gaussian and Lorentzian atom-localized components of equation (1), respectively. In other words, *C* = *C*_G_ = *C*_L_, and *f*_*j*_′^local^(*Z*_*j*_, *B*_*j*_, *E*_0_, *s*) = *C*_G_*f*_*j*_′^local^(*Z*_*j*_, *B*_*j*_, *E*_0_, *s*)_G_ + *C*_L_*f*_*j*_′^local^(*Z*_*j*_, *B*_*j*_, *E*_0_, *s*)_L_ in the present context and in relation to equation (1).

**Figure 1:**
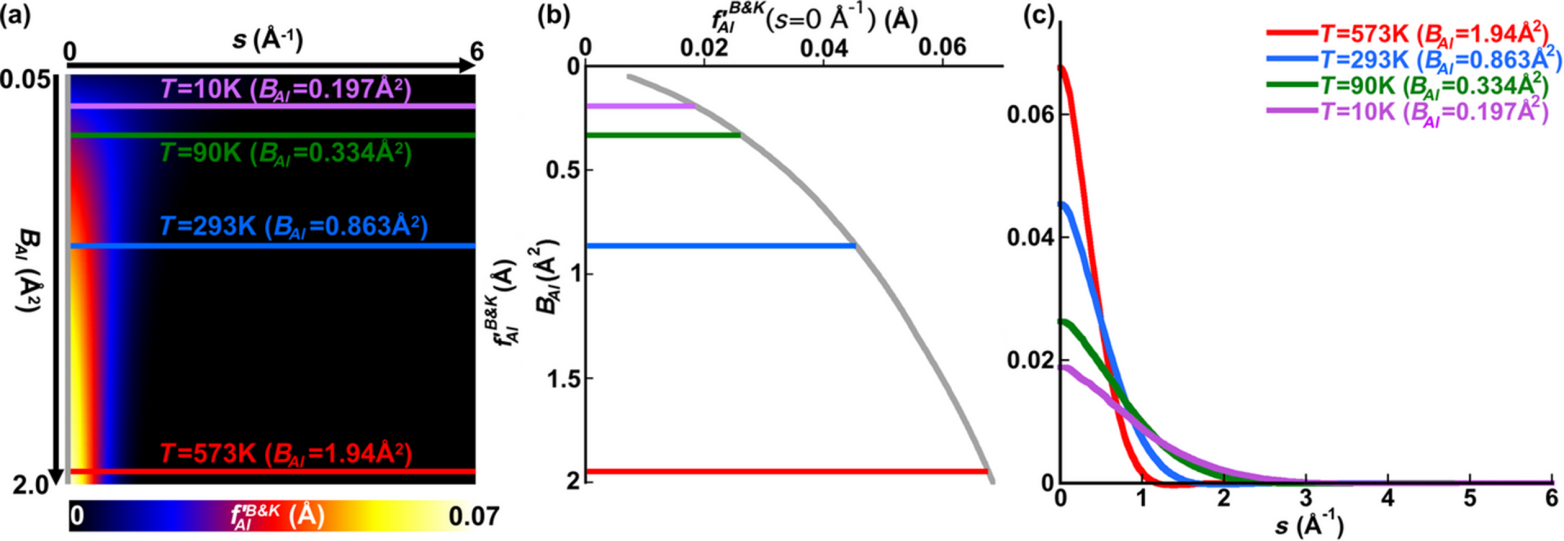
(*a*) The Bird and King absorption factor for aluminium, *f*_Al_′^B&K^, plotted at *E*_0_ = 200 keV over the range 0 Å^−1^ ≤ *s* ≤ 6 Å^−1^ and 0.05 Å^2^ ≤ *B*_Al_ ≤ 2.0 Å^2^, with *s* along the horizontal axis and *B*_Al_ along the vertical axis. The magnitude of *f*_Al_′^B&K^ as computed by the *ATOM* subroutine is given by the colour scale, and this plot is constructed from a grid of 200 × 200 pixels uniformly spanning the full range of *B*_Al_ and *s* specified above. (*b*) A plot of *f*_Al_′^B&K^ as a function of *B*_Al_ at *s* = 0 Å^−1^. The coloured lines in (*a*) and (*b*) correspond to four different temperatures (*T =* 10 K, *T =* 90 K, *T =* 293 K and *T =* 573 K) attainable with liquid helium cooling, liquid nitro­gen cooling, ambient conditions and *in situ* annealing, respectively. (*c*) Profiles of *f*_Al_′^B&K^ as a function of *s* are plotted for each of these four temperatures.

Fig. 1 ▸ plots *f*_Al_′^B&K^(*Z*_Al_ = 13, 0.05 Å^2^ ≤ *B*_Al_ ≤ 2.0 Å^2^, *E*_0_ = 200 keV, 0 Å^−1^ ≤ *s* ≤ 6 Å^−1^), *i.e.* the absorption factor for aluminium with 200 keV incident electrons over the full range of Debye–Waller parameters and *s* calculable by *ATOM*. Aluminium was chosen as the subject of Fig. 1 ▸ for no other reason than that it is the focal element in recent QCBED work by some of the authors (*e.g.* Nakashima *et al.*, 2011 ▸; Nakashima, 2012 ▸; Nakashima, 2017 ▸; Nakashima, 2019 ▸; Tan *et al.*, 2024 ▸). A two-dimensional colour plot of *f*_Al_′^B&K^ as a function of both *s* (horizontal axis) and *B*_Al_ (vertical axis) is shown in Fig. 1 ▸(*a*) and is constructed from a grid of 200 × 200 pixels uniformly spanning the full range of *B*_Al_ and *s* stated above. Five different coloured lines (four horizontal and one vertical) are drawn within this plot. The single vertical (grey) line is the locus of *s* = 0 Å^−1^ from which the plot of *f*_Al_′^B&K^ as a function of *B*_Al_ at *s* = 0 Å^−1^ is obtained as shown in Fig. 1 ▸(*b*). The four horizontal lines correspond to the loci *B*_Al_ = 0.197 Å^2^ (lilac), *B*_Al_ = 0.334 Å^2^ (green), *B*_Al_ = 0.863 Å^2^ (blue) and *B*_Al_ = 1.94 Å^2^ (red), and were chosen because these Debye–Waller parameters correspond to temperatures attainable by liquid helium cooling (*T =* 10 K), liquid nitro­gen cooling (*T =* 90 K), ambient conditions (*T* = 293 K) and *in situ* annealing (*T =* 573 K for aluminium) experiments in TEMs, respectively. It is along these four loci that *f*_Al_′^B&K^ as a function of *s* is plotted in Fig. 1 ▸(*c*). The positions of these lines in terms of *B*_Al_ are also indicated in the plot of *f*_Al_′^B&K^ as a function of *B*_Al_ in Fig. 1 ▸(*b*).

The most notable trend from Fig. 1 ▸ is that, as the temperature and therefore the Debye–Waller parameter increase, the absorption at *s* = 0 Å^−1^ increases while the tail of *f*_Al_′^B&K^ is very rapidly damped as *s* increases. This is readily explained by the increase in TDS with increasing temperature, meaning that the average absorption will increase and thus *f*_Al_′^B&K^(*s* = 0 Å^−1^) will increase. The increased thermal dis­place­ment of atoms at higher temperatures means that the Debye–Waller factor, 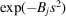 for all atoms *j*, becomes rapidly smaller with increasing *s* due to the larger value of *B_j_* at higher temperatures, thus damping *f*_Al_′^B&K^ very rapidly as *s* increases. *ATOM*-computed absorption factors, *f*_Al_′^B&K^, already include the Debye–Waller factor (Bird & King, 1990 ▸). Furthermore, *f*_*j*_′^B&K^(*Z*_*j*_, *B*_*j*_, *E*_0_, *s*) < 0 for some intermediate values of *s*. This can be seen on close inspection of Fig. 1 ▸(*c*), especially for the case of *B*_Al_ = 1.94 Å^2^. This is a product of the Einstein model and was also evident in earlier work for other elements (*e.g.* Humphreys & Hirsch, 1968 ▸). This happens with *f*_*j*_′^B&K^(*Z*_*j*_, *B*_*j*_, *E*_0_, *s*) for many cases, and although the magnitudes of the dips below 0 are small they are not physically realistic. The development of the function for *f*_*j*_′^local^(*Z*_*j*_, *B*_*j*_, *E*_0_, *s*) in this paper must avoid situations in which *f*_*j*_′^local^(*Z*_*j*_, *B*_*j*_, *E*_0_, *s*) < 0. From Fig. 1 ▸(*b*), it is also evident that *f*_Al_′^B&K^(*s* = 0 Å^−1^) is approximately proportional to 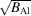. This is informative in the choice of function to be used for *f*_*j*_′^local^(*Z*_*j*_, *B*_*j*_, *E*_0_, *s*), with the aim of closely approximating *f*_*j*_′^B&K^(*Z*_*j*_, *B*_*j*_, *E*_0_, *s*).

The next step is to determine a suitable form for *f*_*j*_′^local^(*Z*_*j*_, *B*_*j*_, *E*_0_, *s*). By experimenting with different combinations of Gaussians and Lorentzians, it was found that the best fit to the plot of *f*_Al_′^B&K^(*Z*_Al_ = 13, 0.05 Å^2^ ≤ *B*_Al_ ≤ 2.0 Å^2^, *E*_0_ = 200 keV, 0 Å^−1^ ≤ *s* ≤ 6 Å^−1^) in Fig. 1 ▸ was obtained if one sets
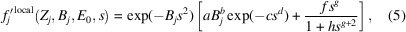
where *a, b, c, d, f, g* and *h* are treated as parameters. Examining this function closely, the first term in the square brackets is Gaussian in terms of *s*, and this term will dominate when *s* is small because the Lorentzian second term vanishes as *s* approaches 0. The multiplier *B_j_^b^* is included in the Gaussian term as there is an obvious dependence of *f*_*j*_′^B&K^(*Z*_*j*_, *B*_*j*_, *E*_0_, *s* = 0 Å^−1^) on *B_j_^b^* in Fig. 1 ▸, where one might estimate that parameter *b* ≃ 0.5 for the case of aluminium with *E*_0_ = 200 keV, as stated above. The Debye–Waller factor, 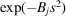, modifies the entire function in the square brackets.

**Figure 2:**
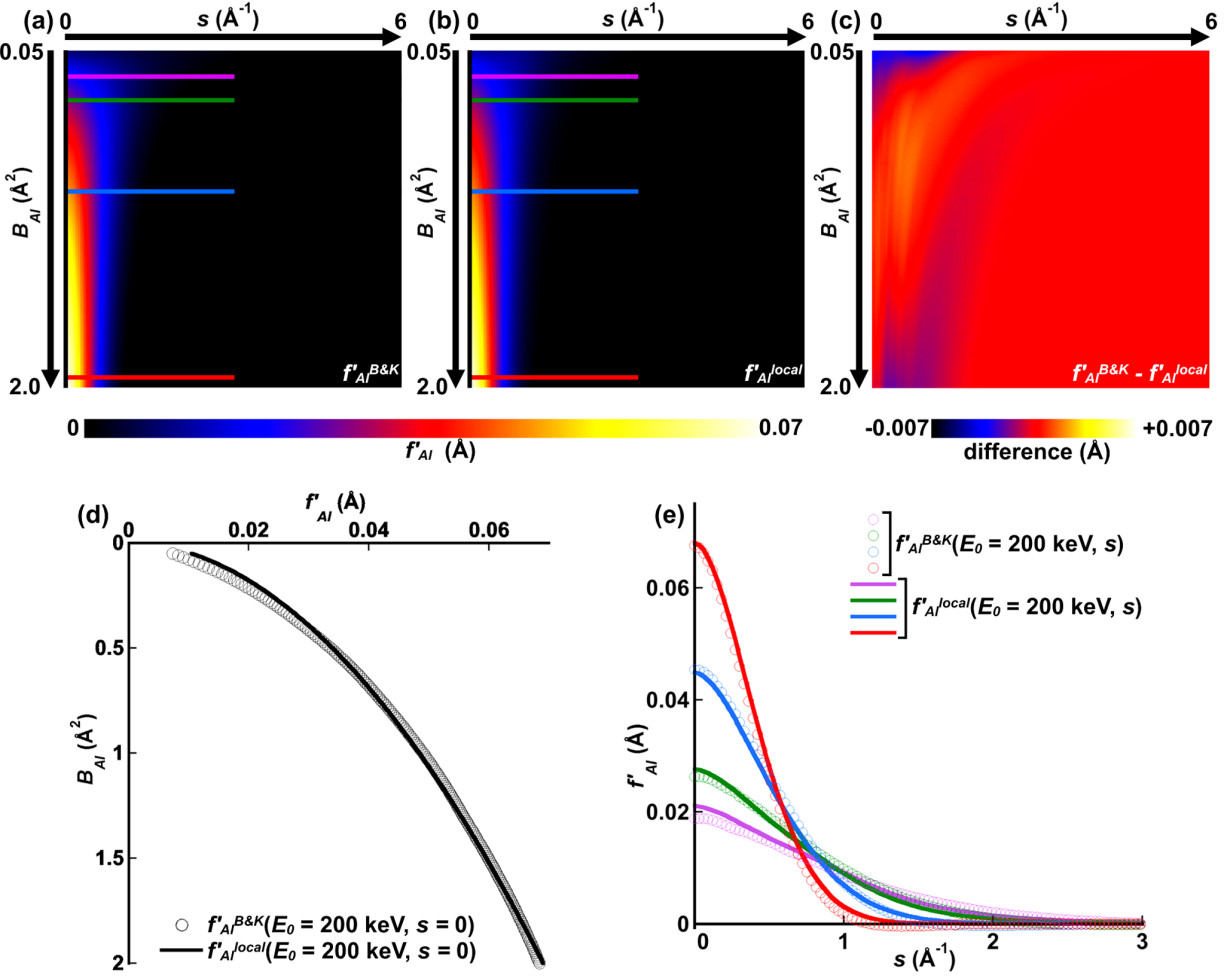
A comparison of (*a*) *f*_Al_′^B&K^(*Z*_Al_ = 13, 0.05 Å^2^ ≤ *B*_Al_ ≤ 2.0 Å^2^, *E*_0_ = 200 keV, 0 Å^−1^ ≤ *s* ≤ 6 Å^−1^) and (*b*) *f*_Al_′^local^(*Z*_Al_ = 13, 0.05 Å^2^ ≤ *B*_Al_ ≤ 2.0 Å^2^, *E*_0_ = 200 keV, 0 Å^−1^ ≤ *s* ≤ 6 Å^−1^) after fitting equation (5) to the former. The parameters producing the best fit of equation (5) to *f*_Al_′^B&K^ calculated by the *ATOM* subroutine are *a* = 0.048395, *b* = 0.51240, *c* = 1.49948, *d* = 1.69455, *f* = 0.0122095, *g* = 2.00334 and *h* = 0.8985707. The difference map (*c*) is shown with a colour scale that spans magnitudes ten times smaller than those in the individual images of *f*_Al_′^B&K^ and *f*_Al_′^local^. Graph (*d*) compares *f*_Al_′^B&K^ and *f*_Al_′^local^ along the black locus in (*a*) and (*b*) at *s* = 0 [as in Figs. 1 ▸(*a*) and 1 ▸(*b*)], and graph (*e*) compares *f*_Al_′^B&K^ and *f*_Al_′^local^ along the coloured loci at *B*_Al_ corresponding to *T =* 10 K, *T =* 90 K, *T =* 293 K and *T =* 573 K as in Fig. 1 ▸(*c*), but only over the range 0 Å^−1^ ≤ *s* ≤ 3 Å^−1^ as values beyond *s* = 3 Å are vanishingly small.

Fig. 2 ▸(*a*) repeats Fig. 1 ▸(*a*), which shows *f*_Al_′^B&K^(*Z*_Al_ = 13, 0.05 Å^2^ ≤ *B*_Al_ ≤ 2.0 Å^2^, *E*_0_ = 200 keV, 0 Å^−1^ ≤ *s* ≤ 6 Å^−1^) as a function of *B*_Al_ and *s*. Fig. 2 ▸(*b*) shows *f*_Al_′^local^(*Z*_Al_ = 13, 0.05 Å^2^ ≤ *B*_Al_ ≤ 2.0 Å^2^, *E*_0_ = 200 keV, 0 Å^−1^ ≤ *s* ≤ 6 Å^−1^) as a function of *B*_Al_ and *s* after fitting equation (5) to the plot of Fig. 2 ▸(*a*). From the values of the refined parameters listed in the caption to Fig. 2 ▸, the prediction that *b* ≃ 0.5 is satisfied in the present example for aluminium with 200 keV electrons. Furthermore, *g* ≃ 2 suggests that the Lorentzian term in equation (5) has even parity, which is a natural consequence of the radial symmetry expected from the absorption processes being accounted for.

Fig. 2 ▸(*c*) shows a map of the difference between Figs. 2 ▸(*a*) and 2 ▸(*b*), *i.e.f*_Al_′^B&K^ − *f*_Al_′^local^. The mismatch is at least an order of magnitude smaller than the individual magnitudes of *f*_Al_′^B&K^ and *f*_Al_′^local^. In the process of optimizing the fit between *f*_Al_′^B&K^ and *f*_Al_′^local^, a mismatch parameter was minimized, which is defined as
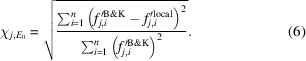
This is the root mean square (RMS) fractional difference between *f*_*j*_′^B&K^ and *f*_*j*_′^local^, where the sums are over the *i* = 1 to *n* pixels that make up both images for element *j* with beam electrons of energy *E*_0_ in the generalized case. For the present example of aluminium and 200 keV beam electrons, 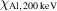
 = 0.041 for Figs. 2 ▸(*a*) and 2 ▸(*b*), and the difference between them is shown in Fig. 2 ▸(*c*).

Fig. 2 ▸(*d*) plots *f*_Al_′^B&K^ and *f*_Al_′^local^ as functions of *B*_Al_ along the locus *s =* 0 Å^−1^ (black), and Fig. 2 ▸(*e*) shows both *f*_Al_′^B&K^ and *f*_Al_′^local^ plotted as functions of *s* for the same values of *B*_Al_ examined in Fig. 1 ▸ but over the reduced range of 0 Å^−1^ ≤ *s* ≤ 3 Å^−1^, for the sake of magnifying the differences between *f*_Al_′^B&K^ and the fitted *f*_Al_′^local^ at low values of *s*. From all of these plots, it appears that equation (5) with best-fit refined parameters *a* to *h* is a very close approximation to the *ATOM* subroutine and thereby the Bird and King model for phenomenological absorption localized to each atom.

**Figure 3:**
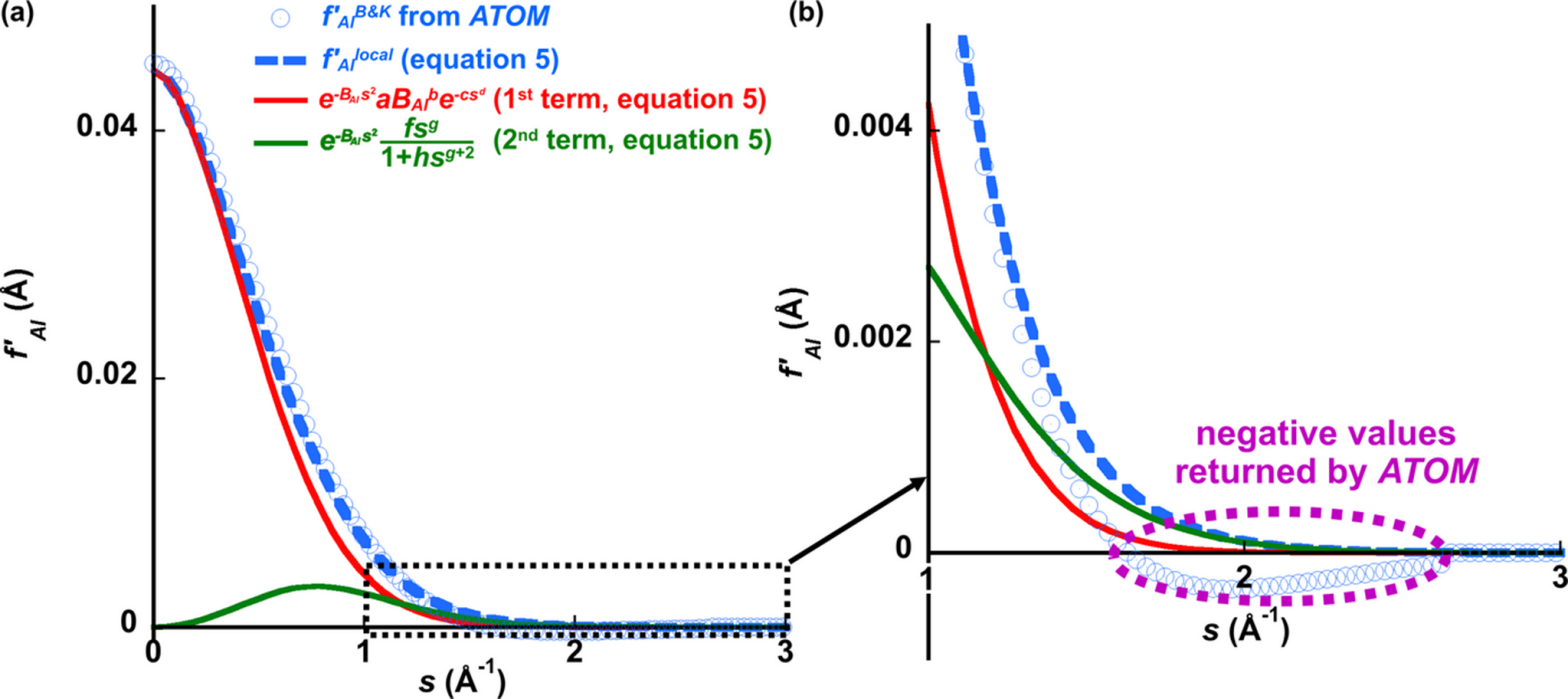
(*a*) Plots of *f*_Al_′^B&K^ and the fitted *f*_Al_′^local^ showing the contributions of each term in equation (5). (*b*) Magnifying the region 1 Å^−1^ ≤ *s* ≤ 3 Å^−1^ in (*a*) shows that the *f*_Al_′^B&K^ values calculated by the *ATOM* subroutine are negative at intermediate values of *s*. Equation (5), which constitutes *f*_*j*_′^local^and approximates *f*_*j*_′^B&K^, is always positive. In the present case of aluminium (*j* = Al) and beam electrons with *E*_0_ = 200 keV, the Lorentzian component dominates *f*_Al_′^local^ for values of *s* > 1.2 Å^−1^.

Using the values of the best-fit parameters in the caption of Fig. 2 ▸, the relative contributions and forms of the terms in equation (5) can be examined. This is done in Fig. 3 ▸, where the blue plot in Fig. 2 ▸(*e*), corresponding to ambient temperature (*T* = 293 K and *B*_Al_ = 0.863 Å^2^), is decomposed into its different components as per equation (5).

At least for this case (aluminium at room temperature with 200 keV beam electrons), it appears that the Gaussian term is dominant for the lower values of *s*, which, in terms of elastic scattering, includes the components of the electrostatic potential distribution associated with bonding. This suggests that techniques like QCBED, which measures the bonding-sensitive elastic structure factors of the crystal potential, can reasonably approximate *f*_*j*_′^local^ with just a Gaussian term. In fact, QCBED using small Laue circle geometries is likely to be much less sensitive to the Lorentzian component of *f*_Al_′^local^ given in equation (5).

Accepting equation (5) as a suitable and likely form for *f*_*j*_′^local^(*Z*_*j*_, *B*_*j*_, *E*_0_, *s*), it is important to establish whether the number of refineable variables can be reduced by replacing them with functions of the variables *Z*_*j*_, *B*_*j*_, *E*_0_ and *s*. As a first test, in the same manner as was done for aluminium and 200 keV electrons in Fig. 2 ▸, the fitting of equation (5) to *f*_*j*_′^B&K^(*Z*_*j*_, *B*_*j*_, *E*_0_, *s*) from the *ATOM* subroutine was repeated for Al (*Z =* 13), Cu (*Z =* 29), Ag (*Z* = 47), Nd (*Z* = 60), Au (*Z =* 79) and U (*Z* = 92), and, in each of these cases, with beam electron energies in the range 1 keV ≤ *E*_0_ ≤ 1 MeV. In other words, this meant that optimized sets of parameters and their associated RMS misfits {*a, b, c, d, f, g, h*, 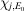} were obtained as a function of both *Z_j_* and *E*_0_ from comparisons of *f*_Al_′^B&K^(*Z*_*j*_, 0.05 Å^2^ ≤ *B*_Al_ ≤ 2.0 Å^2^, *E*_0_, 0 Å^−1^ ≤ *s* ≤ 6 Å^−1^) and *f*_Al_′^local^(*Z_j_*, 0.05 Å^2^ ≤ *B*_Al_ ≤ 2.0 Å^2^, *E*_0_, 0 Å^−1^ ≤ *s* ≤ 6 Å^−1^) for each of the elements given above, for 1 keV ≤ *E*_0_ ≤ 1 MeV. The only difference in procedure for these refinements was that a 100 × 100 grid of pixels spanning 0.05 Å^2^ ≤ *B_j_* ≤ 2.0 Å^2^ and 0 Å^−1^ ≤ *s* ≤ 6 Å^−1^ was used instead of 200 × 200 pixels as in Figs. 1 ▸ and 2 ▸.

**Figure 4:**
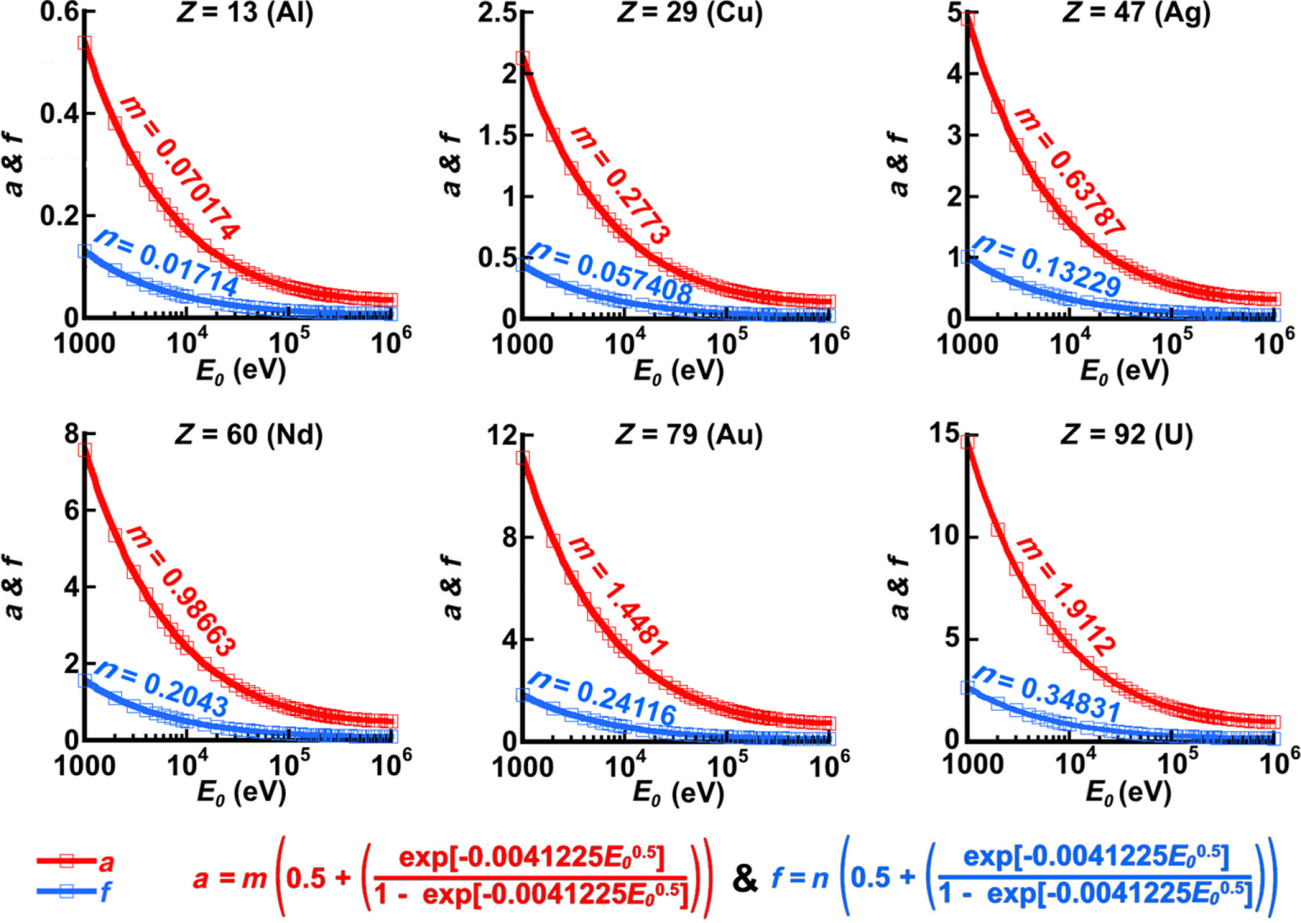
Graphs of parameters *a* and *f* in equation (5) (shown as red and blue squares, respectively) versus the beam electron energy, *E*_0_, after fitting equation (5) to *f*_*j*_′^B&K^(*Z_j_*, 0.05 Å^2^ ≤ *B*_*j*_ ≤ 2.0 Å^2^, *E*_0_, 0 Å^−1^ ≤ *s* ≤ 6 Å^−1^) for Al (*Z =* 13), Cu (*Z =* 29), Ag (*Z* = 47), Nd (*Z* = 60), Au (*Z =* 79) and U (*Z* = 92). The fitted functions for *a* versus *E*_0_ and *f* versus *E*_0_ (red and blue lines, respectively) all have the same form (given explicitly below the plots), independent of the element and independent of the parameter *a* or *f*. The only difference is in the relative magnitudes of the functions, so these are assigned new parameters *m* and *n* for the functions describing *a* and *f*, respectively. The functions for *a* and *f* versus *E*_0_ fit the plotted points perfectly without any mismatch at all.

In performing these fits, it transpired that the parameters *b, c, d, g* and *h* were completely independent of *E*_0_ and only dependent on *Z_j_*, remaining constant during each refinement of equation (5) in fitting *f*_Al_′^B&K^(*Z_j_*, 0.05 Å^2^ ≤ *B*_Al_ ≤ 2.0 Å^2^, *E*_0_, 0 Å^−1^ ≤ *s* ≤ 6 Å^−1^) for any one element *Z_j_*. Only the parameters *a* and *f* are dependent on *E*_0_, and their dependence on electron energy is plotted in Fig. 4 ▸ for each of the six elements given above.

As becomes clear from Fig. 4 ▸, parameters *a* and *f* in equation (5) have the same form when plotted as a function of *E*_0_, independent of atomic number. Their magnitudes increase with increasing atomic number. A variety of asymptotic functions were tested in fitting *a* and *f* versus *E*_0_, with the following form yielding perfect fits in every case:
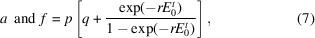
where *p, q, r* and *t* are introduced as refinable variables in fitting equation (7) to the points in the plots of *a* and *f* versus *E*_0_ for each of the elements shown in Fig. 4 ▸. Thus, 12 fits were carried out, and, in all cases, the parameters *q, r* and *t* were found to be constant between different elements and between *a* and *f* versus *E*_0_. It turned out that *q = t = *0.5 and *r* = 0.0041225 in all cases, and that only *p* changed from element to element and between *a* and *f*. One can therefore express *a* and *f* as follows:
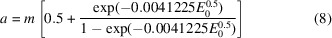
and
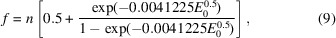
where *m* and *n* are retained as variable parameters that simply allow for different magnitudes of these functions, as observed in Fig. 4 ▸.

Substituting equations (8) and (9) into equation (5) builds in the energy dependence of *f*_*j*_′^local^(*Z*_*j*_, *B*_*j*_, *E*_0_, *s*), and equation (5) becomes
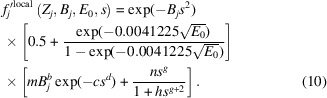


The next step is to determine the dependence of the variable parameters *m, n, b, c, d, g* and *h* in equation (10) on atomic number, *Z_j_*. Given that the dependence of *f*_*j*_′^local^(*Z*_*j*_, *B*_*j*_, *E*_0_, *s*) on *E*_0_ has already been dealt with completely, the electron energy for all future analyses is set to *E*_0_ = 200 keV. The same approach as described in Fig. 2 ▸ and the surrounding text is now taken for all elements accommodated by the *ATOM* subroutine, resulting in a comparison between *f*_*j*_′^B&K^(*Z_j_*, 0.05 Å^2^ ≤ *B_j_* ≤ 2.0 Å^2^, *E*_0_ = 200 keV, 0 Å^−1^ ≤ *s* ≤ 6 Å^−1^) and *f*_*j*_′^local^(*Z_j_*, 0.05 Å^2^ ≤ *B_j_* ≤ 2.0 Å^2^, *E*_0_ = 200 keV, 0 Å^−1^ ≤ *s* ≤ 6 Å^−1^) for *Z_j_* = 1 to 98 (H to Cf). Again, as with the examination of the dependence of *f*_*j*_′^local^(*Z*_*j*_, *B*_*j*_, *E*_0_, *s*) on *E*_0_, the only difference in procedure for these comparisons from that shown in Fig. 2 ▸ was that a 100 × 100 grid of pixels spanning 0.05 Å^2^ ≤ *B_j_* ≤ 2.0 Å^2^ and 0 Å^−1^ ≤ *s* ≤ 6 Å^−1^ was used instead of 200 × 200 pixels as was used for Figs. 1 ▸ and 2 ▸.

For each element *j*, an optimal set of parameters and associated RMS misfit were returned, *i.e.* {*m_j_, n_j_, b_j_, c_j_, d_j_, g_j_*, *h_j_*, 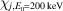}. An overall assessment of the ability of equation (10) to approximate *f*_*j*_′^B&K^(*Z_j_*, 0.05 Å^2^ ≤ *B_j_* ≤ 2.0 Å^2^, *E*_0_ = 200 keV, 0 Å^−1^ ≤ *s* ≤ 6 Å^−1^) – in other words, the ability of equation (10) to approximate the entirety of the *ATOM* subroutine – can be gained by considering the mean RMS misfit, 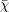, averaged over all of the elements considered. With all seven parameters – *m, n, b, c, d, g* and *h* – allowed to vary, 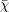
 = 0.0407 ± 0.0007. This figure can be regarded as the benchmark RMS misfit with all degrees of freedom in equation (10) available. As each of the parameters – *m, n, b, c, d, g* and *h* – is replaced, the performance of equation (10) in approximating *f*_*j*_′^B&K^(*Z*_*j*_, *B*_*j*_, *E*_0_, *s*) is expected to deteriorate.

**Figure 5:**
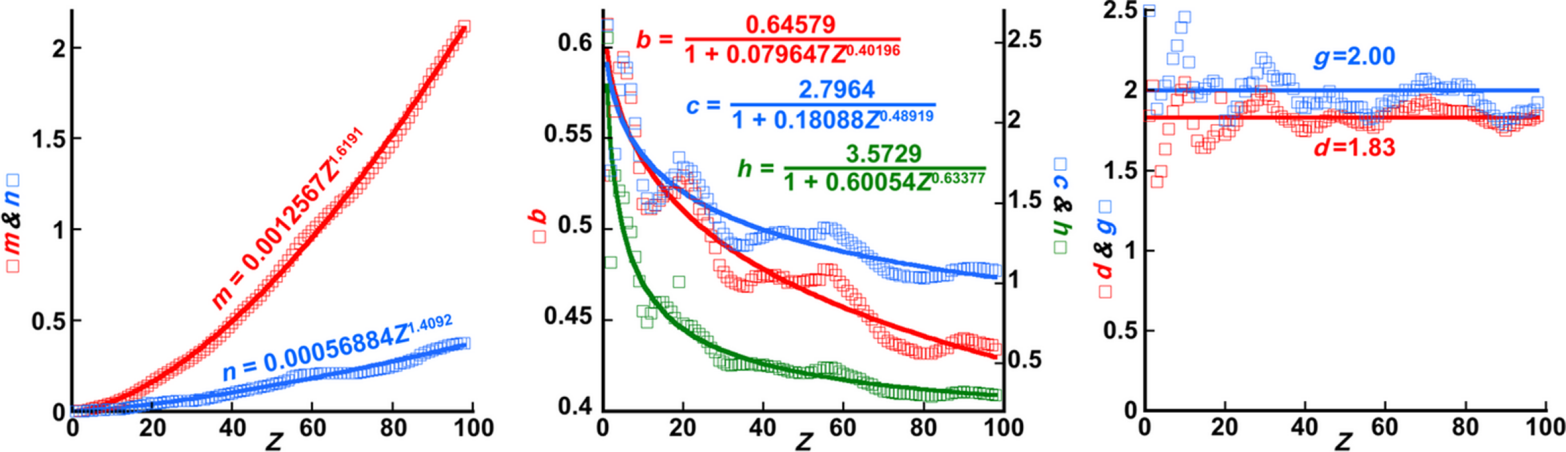
Plots of the variable parameters *m, n, b, c, d, g* and *h* (shown as coloured squares with colour coding indicated on the vertical axes) versus *Z* after fitting equation (10) to *f*_*j*_′^B&K^(*Z_j_*, 0.05 Å^2^ ≤ *B_j_* ≤ 2.0 Å^2^, *E*_0_ = 200 keV, 0 Å^−1^ ≤ *s* ≤ 6 Å^−1^) obtained from the *ATOM* subroutine. Parameters have been grouped into plots on the basis of similar behaviours with respect to *Z*. In every case, functions have been found that best fit the refined values of each of the parameters as a function of *Z* (solid lines in each of the plots) and these are written explicitly into each plot.

The optimal parameters {*m_j_, n_j_, b_j_, c_j_, d_j_, g_j_*, *h_j_*} for each element are plotted as a function of *Z_j_* in Fig. 5 ▸. The parameters are grouped into each of the graphs on the basis of similar behaviours with respect to *Z*, and functions have been found that give the best fit to the plot of each parameter.

Parameters *m* and *n* are fitted quite well by the function
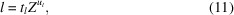
where *l = m* or *n*, and *t_l_* and *u_l_* are refined in the fit of equation (11) to *m* and *n* versus *Z*. Parameters *b, c* and *h* in equation (10) have general trends in terms of *Z* that can be fitted with
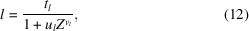
where *l = b, c* or *h*, and the refinable fit parameters are *t_l_, u_l_* and *v_l_*. The result of each fit of equation (11) to *m* and *n* versus *Z* is written into the first graph in Fig. 5 ▸ for the red and blue sets, respectively, whilst the result of each fit of equation (12) to *b, c* and *h* versus *Z* is written into the second graph in Fig. 5 ▸ for the red, blue and green sets, respectively. Parameters *d* and *g* from equation (10) were fitted with constants, *d =* 1.83 and *g =* 2.00, as shown in the third graph in Fig. 5 ▸. That *g* is 2 is rather unsurprising because this confirms the expected even parity of the Lorentzian component in *f*_*j*_′^local^(*Z*_*j*_, *B*_*j*_, *E*_0_, *s*) as a function of *s*.

The plots of the individual points determined by fitting equation (10) to *f*_*j*_′^B&K^(*Z_j_*, 0.05 Å^2^ ≤ *B_j_* ≤ 2.0 Å^2^, *E*_0_ = 200 keV, 0 Å^−1^ ≤ *s* ≤ 6 Å^−1^) for each element *j* show varying degrees of oscillation in addition to the general trends fitted by equations (11) and (12) in the cases of *m, n, b, c* and *h* and constants in the cases of *d* and *g*. These oscillations are much more pronounced for *b, c, h, d* and *g* than for *m* and *n*. An additional improvement in the fits might be obtained if a sine component was incorporated into each of the fits; however, given that the periods of the oscillations seem to change with increasing *Z* and that the oscillations are not particularly regular in form, it was decided that the replacement functions for all seven parameters – *m, n, b, c, h, d* and *g* – should be kept as simple as possible if this does not result in a large deterioration in the fit of equation (10) to *f*_*j*_′^B&K^(*Z_j_*, 0.05 Å^2^ ≤ *B_j_* ≤ 2.0 Å^2^, *E*_0_ = 200 keV, 0 Å^−1^ ≤ *s* ≤ 6 Å^−1^) averaged over all elements *j*.

**Figure 6:**
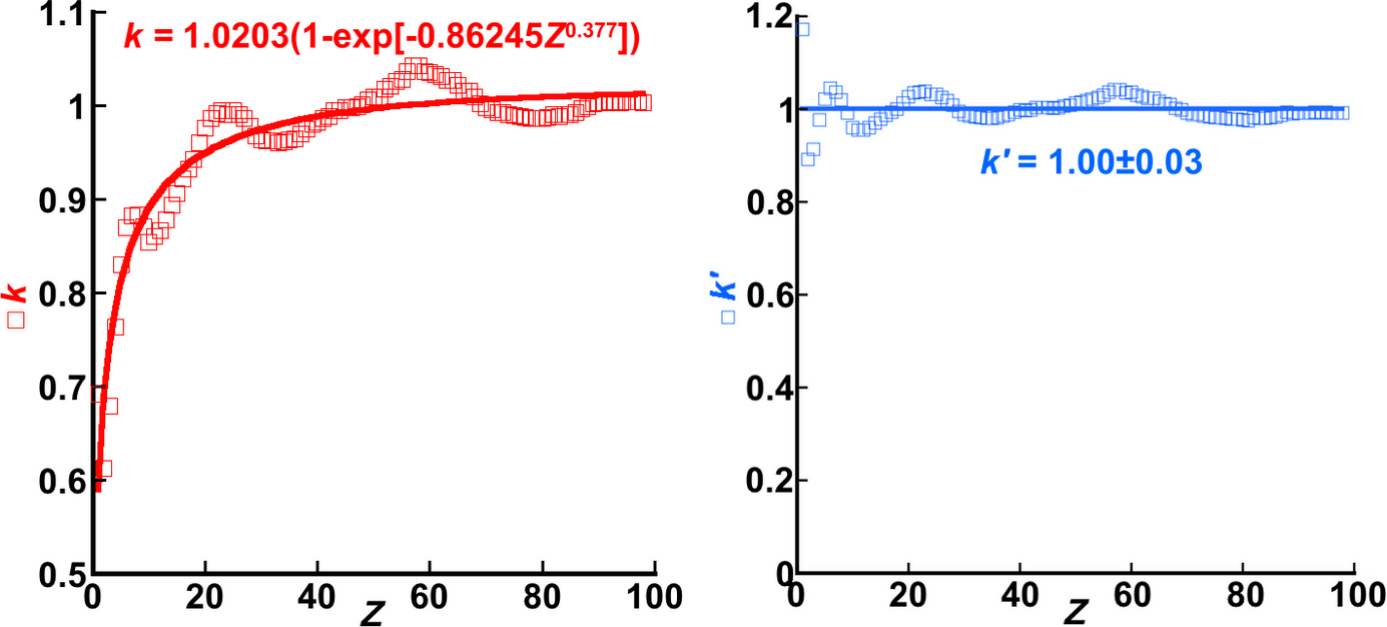
Plots of the proportionality constants *k* in equation (13) and *k′* in equation (15) (red and blue coloured squares, respectively) versus *Z*. The general trend in *k* with respect to *Z* is fitted by equation (14) (solid red line), yielding the values of the parameters written into the equation in the plot. In contrast, *k′* is seen to oscillate about 1 (solid blue line).

Replacing *m, n, b, c, h, d* and *g* in equation (10) with the fitted functions detailed above results in
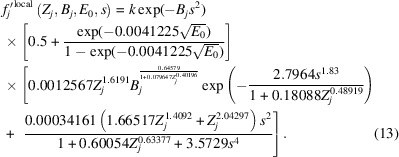
A proportionality constant, *k*, has been introduced as a means of fitting equation (13) to *f*_*j*_′^B&K^(*Z_j_*, 0.05 Å^2^ ≤ *B_j_* ≤ 2.0 Å^2^, *E*_0_ = 200 keV, 0 Å^−1^ ≤ *s* ≤ 6 Å^−1^) for each element *j* in order to check how well the function above approximates the entire *ATOM* subroutine. Repeating the fitting process for all elements returned 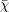
 = 0.044 ± 0.006, which is only a very slight increase in overall mismatch compared with the performance of equation (10) where 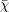 = 0.0407 ± 0.0007 with seven variable parameters. Although the value of *k* was expected to be constant and close to 1 for all elements, this proportionality parameter added for the sake of the fitting process was seen to vary with respect to atomic number *Z*. This is shown in Fig. 6 ▸. This parameter is likely to be absorbing errors incurred by the substitution of parameters *m* and *n* in equation (10) with the fitted functions whose forms are given in equation (11) because the fits (as seen in Fig. 5 ▸) were by no means perfect.

The form of the function fitted to *k* versus *Z* is
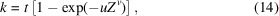
where *t, u* and *v* are fitting parameters whose final values are given in the left-most plot in Fig. 6 ▸. Substitution of this result into equation (13) returns
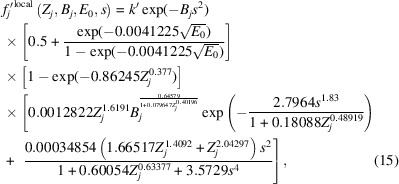
with the value of *t* = 1.0203 absorbed into the coefficients of the two terms in the final set of square brackets of equation (15). Again, a proportionality constant, *k′*, is retained to test equation (15) against *f*_*j*_′^B&K^(*Z_j_*, 0.05 Å^2^ ≤ *B_j_* ≤ 2.0 Å^2^, *E*_0_ = 200 keV, 0 Å^−1^ ≤ *s* ≤ 6 Å^−1^) from the *ATOM* subroutine. This time, the graph of *k′* versus *Z* simply oscillates about *k′ =* 1, as seen in the plot on the right in Fig. 6 ▸. As with previous parameters fitted with equations, the oscillations are not dealt with, and it is at this point that the determination of *f*_*j*_′^local^(*Z*_*j*_, *B*_*j*_, *E*_0_, *s*) concludes.

## 3. Discussion

Revisiting equation (1) in the *Introduction*[Sec sec1], substitution of equation (15) for both of the local terms gives
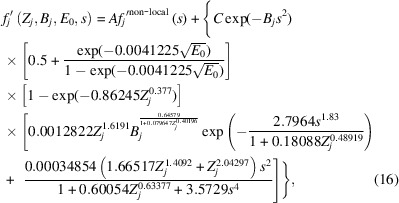
where the parameter *k′* in equation (15) is replaced by parameter *C* = *C*_G_ = *C*_L_ in keeping with equation (1). At this point, the non-local component is replaced by a Dirac delta function in *s*:
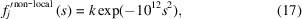
where the multiplier of 10^12^ in the exponential is arbitrarily chosen just because it is a large number with respect to the range of *s* over which *f_j_′*(*Z*_*j*_, *B*_*j*_, *E*_0_, *s*) is considered significant. The expression involves a proportionality constant, *k*, which can be absorbed into the parameter *A* in equation (16). Using a Dirac delta function for *f*_*j*_′^non-local^(*s*) makes the approximation that the sum of all the non-local contributions to the phenomenological absorption can be considered as adding a constant to *f_j_′*(*Z*_*j*_, *B*_*j*_, *E*_0_, *s* = 0). This approximation is equivalent to saying that the non-local contributions are uniformly distributed at all distances from the atoms in real space.

Substituting equation (17) into equation (16) yields:
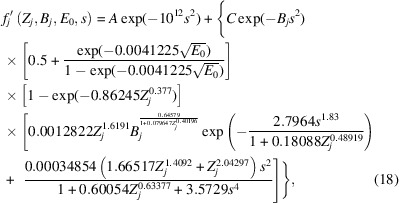
with *k* from equation (17) absorbed into parameter *A* in this equation. This represents a two-parameter model for all phenomenological absorption that can be refined by an experimental technique such as QCBED. However, if one were to apply equation (18) in its present form, the values of *A* and *C* obtained from refinements would not provide a direct indication of the relative contributions of *f*_*j*_′^non-local^(*Z*_*j*_, *B*_*j*_, *E*_0_, *s*) and *f*_*j*_′^local^(*Z*_*j*_, *B*_*j*_, *E*_0_, *s*). This can be seen if one considers *f_j_′*(*Z*_*j*_, *B*_*j*_, *E*_0_, *s* = 0):
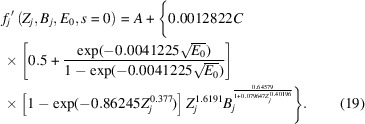
The parameter *C* is modified by a factor that is dependent on *Z_j_, B_j_* and *E*_0_, whilst *A* is not. Therefore equation (18) must be factorized in a way where both *A* and *C* are modified by the same factors that are independent of *s*. This factorization transforms equation (18) into
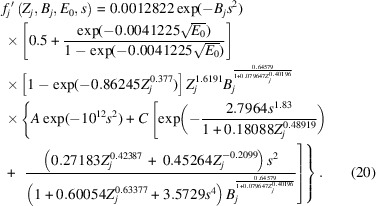
In this form, at *s* = 0,
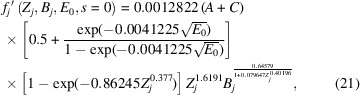
and therefore, the magnitudes of *A* and *C* obtained experimentally are directly comparable and representative of the relative magnitudes of the non-local and local contributions to phenomenological absorption, respectively.

Finally, by segmenting equation (20) into Gaussian and Lorentzian contributions as per equation (1), the following is obtained:
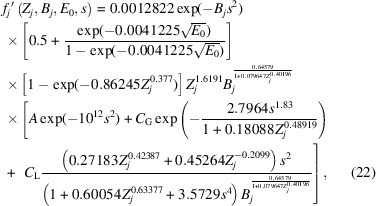
where *C*_G_ and *C*_L_ are variable coefficients for the Gaussian and Lorentzian contributions, respectively, as per the *Introduction*[Sec sec1]. Setting the coefficients to *A* = 0 and *C*_G_ = *C*_L_ = 1 returns the closest fit to the output from the *ATOM* subroutine by Bird & King (1990 ▸).

The central aim was to produce a function that mimics the Bird & King (1990 ▸) inelastic scattering factors (TDS only) as a starting point, but that can be adjusted via a small number of parameters to accommodate contributions from plasmon scattering to QCBED of unfiltered CBED data. Equation (22) is the outcome of this aim. The physical meaning behind each of the terms developed in the equation is purely heuristic as only a parametrized functional mimic of the *ATOM* subroutine was sought. This work was not motivated by improvements to computational speed because this part of QCBED is negligible in terms of computational cost compared with the determinations of diffracted intensities from the many-beam electron scattering equations.

Unfiltered CBED patterns contain a slowly varying diffuse background, which is eliminated by differential QCBED. It is precisely because of its absence from differentiated data that it is desirable to take it into account by adding the Dirac delta function, because this provides two options when it comes to normalizing the simulated differential CBED patterns to the experimental ones:

(i) setting the *A* coefficient in equation (22) to 0 and using equal counts or minimum misfit for normalization, or

(ii) setting the normalization factor in QCBED to a pre-measured incident intensity (measured in the absence of the specimen) and refining the relative contributions of each of the three terms in equation (22) by including *A*, *C*_G_ and *C*_L_ as refined parameters.

Considering option (ii), it is expected that the magnitude of *A* and its ratio to *C*_G_ and *C*_L_ will differ significantly from QCBED with energy filtering to unfiltered differential QCBED, and this is of interest in itself. For both options above, it is expected that *C*_G_ and *C*_L_ will refine to less than unity when performing unfiltered differential QCBED because of the presence of additional differential signal due to plasmon losses (Nakashima & Muddle, 2010*b* ▸; Egerton, 2011 ▸). This is of particular importance if differential QCBED is to be accurate without energy-filtered data, and this is the main motivation of this paper.

While one can also refine *B_j_*, *E*_0_ and *s* (the latter via refinement of the lattice parameters) in QCBED, should one wish to refine *A*, *C*_G_ and *C*_L_, it is better to fix *B_j_*, *E*_0_ and *s* as constant inputs into equation (22) to avoid parameter correlations. Furthermore, while this work has parametrically mimicked the *ATOM* subroutine, which deals with isotropic Debye–Waller parameters, *B_j_*, a more advanced approach in future would follow those of Peng (1997 ▸) or Weickenmeier & Kohl (1998 ▸) who accommodated anisotropic atomic displacement parameters.

In closing, we note that equation (22) has already been implemented successfully in a small number of QCBED studies (Liu, 2019 ▸; Tan *et al.*, 2024 ▸) involving a multislice-based (Cowley & Moodie, 1957 ▸) QCBED algorithm. Future work aims to produce a sequel to this paper where experimental refinements of coefficients *A*, *C*_G_ and *C*_L_ will be investigated for a compound rather than elemental metals, and for both electron-optically filtered and unfiltered data in both the conventional and differential regimes of QCBED.

## 4. Conclusions

Using Bird and King’s *ATOM* subroutine, the present work has empirically developed a functional approximation to the atom-localized contribution to phenomenological absorption [see equation (15)] and followed this up with an equation that approximates all contributions to absorption [see equation (22)]. This functional approximation can be used to replace the *ATOM* subroutine with a single line of code, but more importantly, it is written in a form where all contributions are separated such that their individual relative magnitudes could be refined using experimental data.

With recent developments that allow TEM data collected without electron-optical energy filtering to be used quantitatively in techniques like QCBED (Nakashima, 2007 ▸; Nakashima & Muddle, 2010*b* ▸; Nakashima *et al.*, 2011 ▸; Nakashima, 2012 ▸; Nakashima, 2017 ▸; Peng & Nakashima, 2017 ▸; Peng & Nakashima, 2019 ▸; Nakashima, 2019 ▸; Peng & Nakashima, 2021 ▸; Tan *et al.*, 2024 ▸), the availability of a fully flexible phenomenological absorption model where different contributions can be refined as independent components becomes important. Incorporating equation (22) into the calculations of scattered intensities reduces the number of assumptions about what can and cannot be absorbed into the calculated versus experimental intensity normalization process (Nakashima & Muddle, 2010*b* ▸). Furthermore, by refining only *C*_G_ and *C*_L_ in dealing with absorption (usually *A* is set to 0 since it is eliminated by the standard normalization process), the number of refined parameters in a QCBED analysis is reduced compared with previous standard practice. The norm in QCBED has been to refine three to six structure factors per pattern, together with their corresponding absorption factors (Nakashima, 2017 ▸). The new approach replaces the refinement of three to six individual absorption factors with the refinement of just *C*_G_ and *C*_L_. This not only reduces the number of refined parameters but is also more canonical since refining *C*_G_ and *C*_L_ in equation (22) adjusts all absorption factors in the scattering equations, not just individual ones.

The application of the presently determined phenomenological absorption function in differential QCBED without energy filtering removes errors incurred by using the Bird and King *ATOM* subroutine alone because the normalization process no longer needs to compensate for the unaccounted components (plasmon-loss electrons). Furthermore, as was pointed out in the example of aluminium, the *ATOM* subroutine often returns negative values for the absorption factor at intermediate values of *s =* sin(θ)/λ. This is not the case for the present function as its form prohibits negative values of *f_j_′*(*Z*_*j*_, *B*_*j*_, *E*_0_, *s*).

So far, equation (22) has been applied to QCBED studies in elemental metals (Liu, 2019 ▸; Tan *et al.*, 2024 ▸). A sequel to the present paper is planned where experimental QCBED refinements of coefficients *A*, *C*_G_ and *C*_L_ in equation (22) will be investigated for a compound, and for both electron-optically filtered and unfiltered data in both the conventional and differential regimes of QCBED.

## Figures and Tables

**Table 1:** Modes of QCBED (left-hand column) and the inelastic contributions to the pattern-matched CBED patterns (top row) The presence of a particular type of inelastic signal is indicated by ‘YES’ while its absence is indicated by ‘NO’.

	TDS signal mimicking the elastic rocking curve	Plasmon signal mimicking the elastic rocking curve	TDS signal contributing to the diffuse background	All signals other than TDS contributing to the diffuse background
Conventional energy-filtered QCBED	YES	NO	YES	NO
Differential QCBED with energy filtering	YES	NO	NO	NO
Differential QCBED without energy filtering	YES	YES	NO	NO
